# Diversities of Dental Practice

**Published:** 1861-07

**Authors:** J. Taft


### DIVERSITIES IN DENTAL PRACTICE.

BY J. TAFT.

OWING to a want of a uniform method of instruction, and ample text-books—the former depending upon the latter to a great extent—dentists have not succeeded in obtaining such a uniformity in practice as would be desirable. Each one has, to a great extent, been compelled to carve out his own course; this is especially manifested in the practice of the profession. It is true that the profession of dentistry is comparatively new. There has been and still is much indecision as to the best method of performing many operations. Every one has, to some extent, been an experimenter, endeavoring to obtain the great desideratum, viz.: perfection of operation. This has been as assiduously pursued by the unlearned in the science of our profession, as by those who have the greatest attainments in this respect; though not with the same results. The former will take up a new idea or mode of practice, and claim for it at once all imaginable merit; while the latter carefully brings out his discoveries and modes, and fully tests them by principles which he knows to be infallible. The former upon making discoveries in art and the manipulations pertaining thereto, is liable to be assuming, and arrogate to himself far more than he should; while the true man of science is always modest and unassuming, whatever discoveries and improvements he may make. The former thinks they are his own creation, or at least his own unassisted productions; while the man of science knows that he is at best only unfolding and bringing to light that which was laid up in the archives of science and eternal principles; that he is only making new applications of principles as old as the world. It is just and proper that any one should feel gratification when he has been the instrument of making new and useful developments.

The diversity of methods in dental practice still exists, though not to the same extent as formerly. In some respects this diversity of practice is unfortunate, in some particulars it may be advantageous. Most persons, upon adopting a particular of practice, think it better than any other; and particularly is this the case if the person has an interest in the matter by way of discovery or production. It is strange with what tenacity many operators will cling to methods with which they are identified, even when far better are presented. This is often illustrated by instances where men refuse to see the imperfections of their methods, or make any possible palliation for them.

Some seize hold upon some particular method or thing, and refuse to employ or even to examine any other. It is always pleasing to see any one hold fast to that which is good, but that should not prevent from taking another which is just as good, or perhaps better. We think the true plan is to take everything that is valuable, and employ each when it is most available.

These diversities in practice exercise a pernicious influence upon patients; they are as apparent to them as even to dentists themselves. The remark is often made by patients, "Why, you fill my teeth differently from my former dentist, or you do this or that in quite a different manner." Every dentist is familiar with such expressions. There are quite different methods of treating such expressions and impressions. Some will remark something in this wise, That there are more good methods than one of performing an operation, or perhaps another performed an operation under very different circumstances, and in very dissimilar cases. Such a course will be pursued by the true professional gentleman. Others will reply to such remarks of a patient, that his is the only proper method of accomplishing the work, or at least it is better than any other, and that others are only efficient as they approximate his method. With one making such remarks, a patient of common sense will become displeased and disgusted. It is thus that odium is brought on the profession.

Perhaps some good has grown out of these *diversities of practice.* Every one, under such circumstances, would seek to bring his own peculiar mode to the utmost degree of perfection; especially when he is to some extent identified with it.

Among those with whom there is a disposition to a free interchange of ideas and opinions, the diversities of practice will be exceedingly valuable.

A crude, shapeless idea, in its passage from one mind to another, may and often does receive a brilliancy, illumination and expansion that will at once transform it from chaos to a thing of beauty and usefulness.

The disadvantages that usually attend diversities in practice might be so controlled and modified as to be almost robbed of their power, and again, the occasion for them becomes continually lessened as the profession arrives nearer the true method of accomplishing its work.

